# A glimpse into evolution and dissemination of multidrug-resistant *Acinetobacter baumannii* isolates in East Asia: a comparative genomics study

**DOI:** 10.1038/srep24342

**Published:** 2016-04-13

**Authors:** Ye Feng, Zhi Ruan, Jianfeng Shu, Chyi-Liang Chen, Cheng-Hsun Chiu

**Affiliations:** 1 Sir Run Run Shaw Hospital, Zhejiang University School of Medicine, Hangzhou, China; 2 Institute of Translational Medicine, Zhejiang University School of Medicine, Hangzhou, China; 3 Molecular Infectious Disease Research Center, Chang Gung Memorial Hospital, Taoyuan, Taiwan; 4 Department of Pediatrics, Chang Gung Memorial Hospital, Chang Gung University College of Medicine, Taoyuan, Taiwan

## Abstract

Clonal dissemination is characteristic of the important nosocomial pathogen *Acinetobacter baumannii*, as revealed by previous multi-locus sequence typing (MLST) studies. However, the disseminated phyletic unit is actually MLST sequence type instead of real bacterial clone. Here we sequenced the genomes of 13 multidrug-resistant (MDR) *A. baumannii* strains from Taiwan, and compared them with that of *A. baumannii* from other East Asian countries. Core-genome phylogenetic tree divided the analyzed strains into three major clades. Among them, one ST455 clade was a hybrid between the ST208 clade and the other ST455 clade. Several strains showed nearly identical genome sequence, but their isolation sources differed by over 2,500 km and 10 years apart, suggesting a wide dissemination of the phyletic units, which were much smaller than the sequence type. Frequent structural variation was detected even between the closely related strains in antimicrobial resistance elements such as AbaRI, class I integron, indicating strong selection pressure brought by antimicrobial use. In conclusion, wide clonal dissemination and frequent genomic variation simultaneously characterize the clinical MDR *A. baumannii* in East Asia.

*Acinetobacter baumannii* has been spreading worldwide as an important nosocomial pathogen due to its high adaptation to the environment and ability to develop multi- or even pan-drug resistance^1^,^2^,^3^. Genetic characterization revealed that *A. baumannii* possesses an extensive arsenal of chromosome- and plasmid-borne resistance genes. Most of these resistance genes can be laterally transferred via mobile genetic elements (MGEs), such as insertion sequence (IS), transposon, integron and genomic island^4^,^5^. Taking *bla*_OXA-23_ for example, it encodes a class D β-lactamase to mediate resistance to carbapenems, currently the largest concern posed by *A. baumannii*^6^,^7^. The most common carrier of *bla*_OXA-23_, IS*Aba1*, and its transposon vector help insert *bla*_OXA-23_ into chromosome and plasmid, thereby giving rise to global dissemination of carbapenem-resistant *A. baumannii* (CRAB)^8^,^9^.

In addition to lateral genetic transfer (LGT), the population of CRAB is characteristic of clonal dissemination as revealed by multi-locus sequence typing (MLST). Here, the concept “clone” does not refer to a real bacterial clone but to the MLST-delineated lineages. Currently two MLST schemes are available for *A. baumannii*. According to MLST-IP scheme (developed by Institute Pasteur), Sequence type 2 (ST2) accounts for the majority of CRAB worldwide, which is also called International clone 2 (IC2)^10^,^11^. When MLST-OD (associated with Oxford Database, the other scheme with higher resolution) is applied, IC2 can be further split into a number of different STs, and a much more diverse profile of CRAB population is depicted^12^.

Recently we reported an outbreak of bacteremia caused by *A. baumannii* in critical patients in Chang Gung Memorial Hospital (CGMH) in Taiwan^13^. Further examination of the clonal relationship by MLST-OD scheme identified the major CRAB clones responsible for this outbreak to be ST455 and ST208^14^. As a pandemic lineage, ST208 has been detected in Asia, Europe and North America^15^,^16^,^17^. There are few reports on ST455 currently: it was firstly reported in Taiwan in 2013 and then in Japan in 2014^18^. In this study, we sequenced the genomes of *A. baumannii* strains sampled from the outbreak and compared them with those of ST208 and ST455 strains from East Asian countries. We found that ST455 was a derivative of ST208 by virtue of chromosomal-scale recombination. Continental dissemination of the phyletic units, which is much smaller than MLST sequence type, was also observed, where the strains isolated from a wide spatial and temporal range showed nearly identical genomes.

## Results

### Phylogenetic relationship by MLST and whole genome sequencing

**Figure 1:**
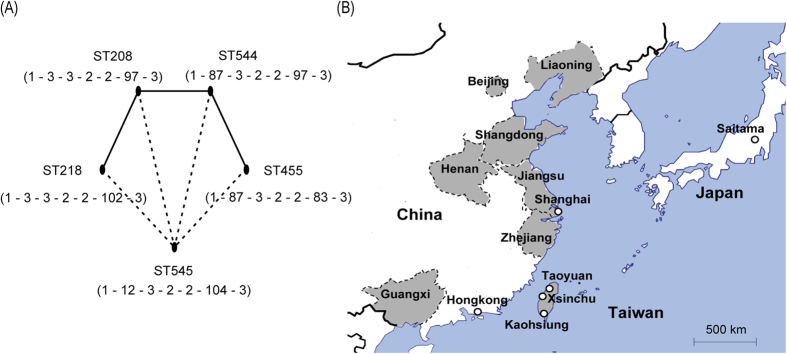
Genetic background and geographical information of the analyzed isolates. Panel (**A**) shows the MLST results, in which the solid lines represent one-allele difference between STs, and the dotted lines represent two-allele difference. The seven numbers under ST represent the combination of alleles used by MLST-OD scheme, that is, *gltA – gyrB – gdhB – recA – cpn60 – gpi – rpoD*. Panel (**B**) shows the collection sites of the analyzed isolates.

**Figure 2:**
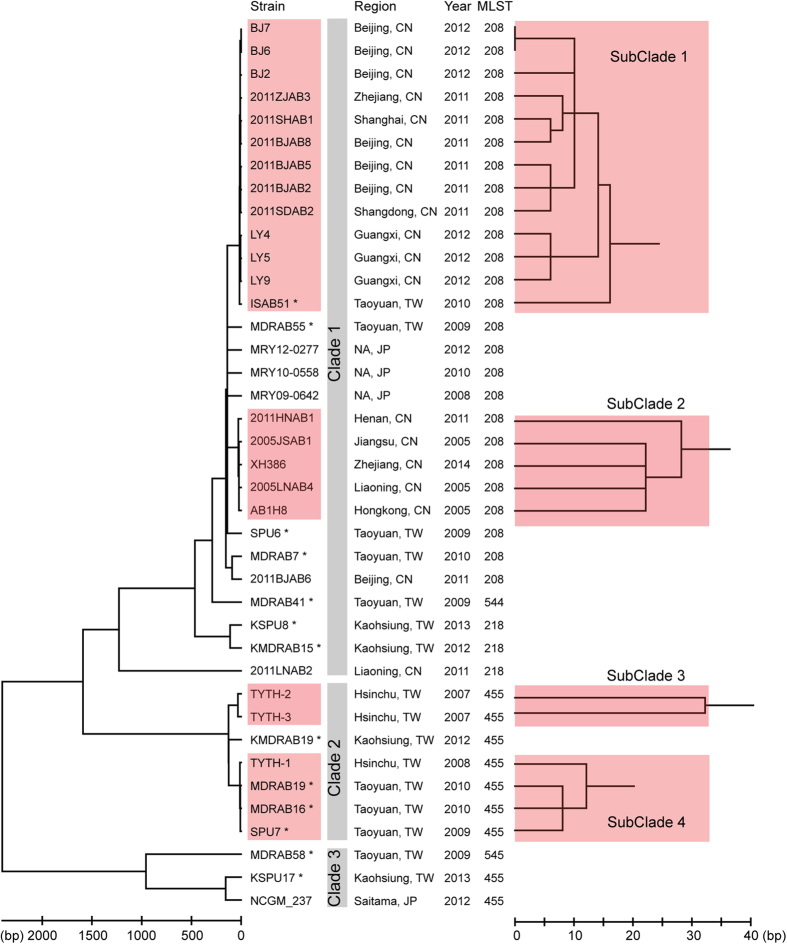
Phylogenetic relationship of the analyzed isolates. The left Neighbor-joining tree is constructed based on the concatenated conserved genes. The scale bar represents the number of different nucleotide bases. At the right, the branches of the four subclades are extended for better resolution. In the middle, the provenance information of the isolates and the MLST-OD typing result are shown. NA, not available. The strains marked by asterisk are sequenced in this study.

Thirteen clinical *A. baumannii* strains, which were collected from CGMH in Taoyuan or Kaohsiung and represented the bacteremic outbreak^13^,^14^, were subjected to whole genome sequencing (WGS). While the MLST-IP scheme classified all strains into ST2, the MLST-OD scheme identified five STs for these strains. The minimum spanning tree analysis indicated that ST218 and ST544 were single locus variants (SLVs) of ST208, and ST455 was further derived from ST544 (Fig. 1A). ST545 linked the above four STs with two different loci. The GenBank and BacWGSTdb^19^ database were searched for *A. baumannii* genomes belonging to these STs, and a total of 26 such strains were found to be collected from East Asia, including Mainland China, Hong Kong, Taiwan, and Japan (Fig. 2).

**Figure 3:**
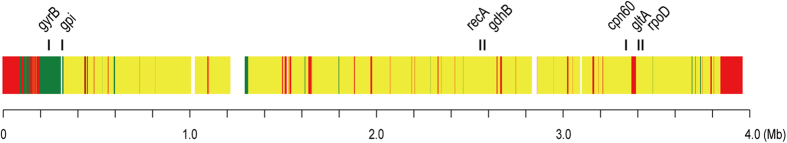
Mosaic structure of Clade 2 genome. Each gene of Clade 2 is compared against its ortholog in Clade 1 and Clade 3. The strains XH386, TYTH-1 and NCGM_237 represent Clade 1, Clade 2 and Clade 3, respectively. Gene order is referred to according to the genome of TYTH-1. Genes in red represent those for which Clade 2 is closer to Clade 1 than Clade 3; genes in green represent those for which Clade 2 is closer to Clade 3 than Clade 1; genes in yellow represent those for which Clade 2 has equal distance to Clade 1 and Clade 3. The positions of seven housekeeping genes used by MLST-OD scheme are marked.

A phylogenetic tree based on single nucleotide polymorphisms (SNPs) within core genome was built in order to reveal a more detailed relationship among the analyzed strains. By comparing their genomes, 2,746 genes were conserved among strains and constituted a 2,525,551-bp concatenated alignment. Although all strains were relatively similar to each other by less than 6,000 SNPs (genetic distance < 0.23%), they were clearly divided into three major clades from the tree (Fig. 2). While ST208 and its SLV ST218 and ST544 constituted Clade 1, ST455 strains were separated into Clade 2 and Clade 3. The strains XH386, TYTH-1 and NCGM_237 had their genomes completely sequenced and thus were chosen to represent the three clades, respectively. The comparative genomic analysis revealed a mosaic structure in the genome of Clade 2 (Fig. 3). In detail, 5.2% of the conserved genes in the genome of Clade 2 were more similar to their orthologs in Clade 3 than to that in Clade 1. Two loci of MLST-OD scheme, *gyrB* and *gpi*, were included in this category so that the Clade 2 strains were assigned to ST455. However, 8.2% of genes in Clade 2 showed a higher identity to Clade 1 than to Clade 3, explaining why Clade 2 was relatively closer to ST208 in the phylogenetic tree. The remaining 86.7% of genes showed identical identity to either Clade 1 or Clade 3. Interestingly, the majority of the genes involved in the mosaic structure were clustered into large blocks, indicating the presence of chromosome-scale recombination.

**Figure 4:**
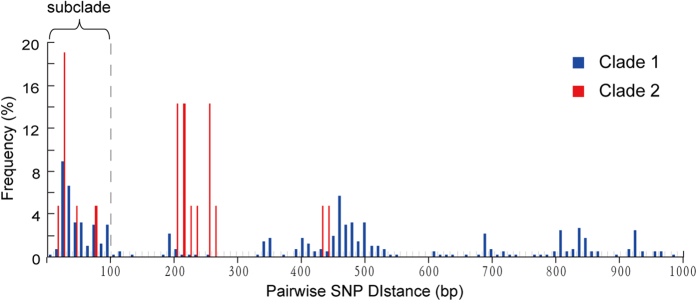
Histogram of pairwise SNP distances. 100 SNPs is used as the cut-off value for defining subclades.

A deeper look inside each of the phylogenetic clades was taken by counting the number of pairwise SNPs within clades (Fig. 4). The first peak centered at the distance of <50 SNPs and finished prior to 100 SNPs. By taking the 100-SNP distance as the cut-off value, four subclades were identified (Fig. 2). Although being genetically close to each other, the strains within the same subclades showed highly diverse isolation sites and time. Taking SubClade 1 and SubClade 2 as an example, the strains came from different provinces of China, which were over 3,000 km apart at most (Figs 1B and 2); the earliest strain was isolated in the year 2005, and the most recent strain was in 2014.

However, strains with the same isolation site and time did not necessarily belong to the same subclades. The four ST208 Taiwan strains were collected from the same hospital during the bacteremic outbreak, and yet they were different from each other by 275 SNPs in average.

### Distribution of *bla*_OXA_ and IS*Aba1* insertion sites

**Figure 5:**
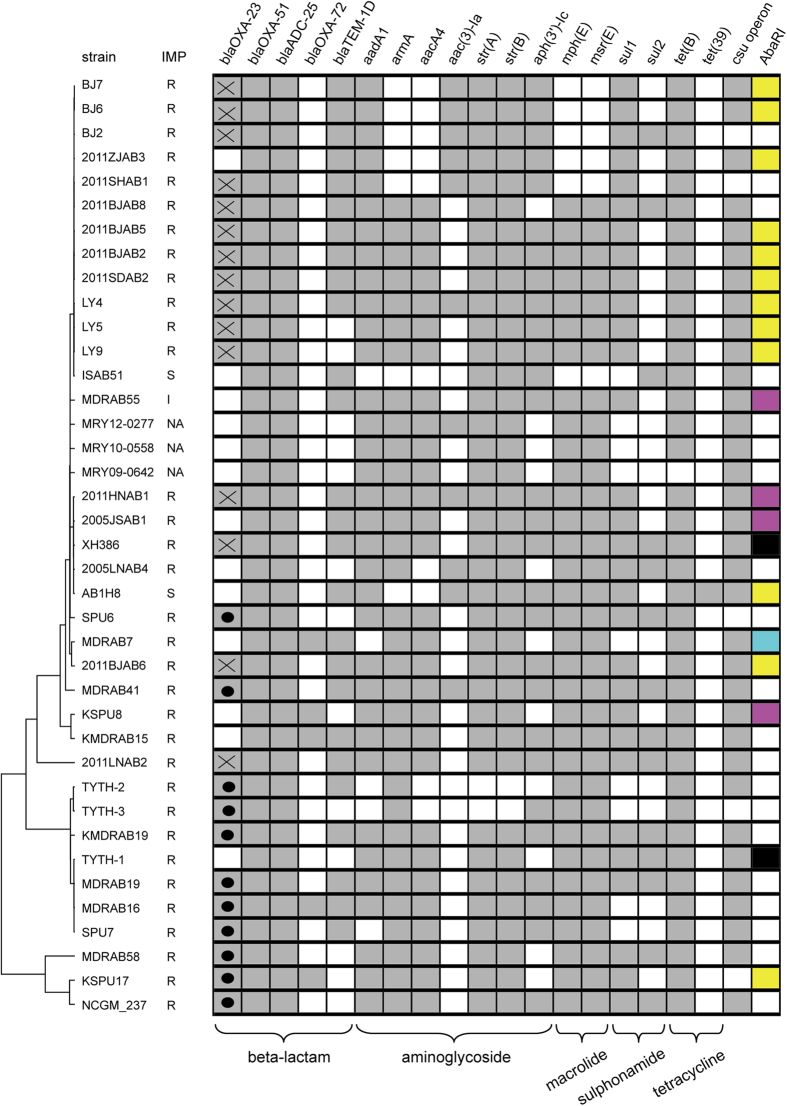
Distribution of resistance genes. The cell in grey indicates the presence of the gene while the blank cell indicates absence of the gene. The dot and cross in the column *bla*_OXA-23_ represent the Tn*2006* and Tn*2009* carrier, respectively. The different AbaRI types are marked by different colors: cyan, type I; yellow, type II; magenta, type III; black, type IV; blank, unknown. The resistance genes of str. TYTH-1 includes those located on chromosome only.

The carbapenemase-encoding gene *bla*_OXA-23_ was found in 27 strains, all of which were resistant to carbapenem. The gene *bla*_OXA-23_ was exclusively located within either Tn*2009* or Tn*2006*. The distribution of the two transposons was intensely associated with geographical locations in this study: Tn*2009* was only identified in Mainland China, and Tn*2006* only in Hong Kong, Japan and Taiwan (Fig. 5). In three strains the imipenem resistance was not mediated by *bla*_OXA-23_ but instead by plasmid-borne *bla*_OXA-72_. The strains NCGM_237 and MDRAB16 carried both *bla*_OXA-23_ and *bla*_OXA-72_ simultaneously. Three strains from Mainland China (str. 2011ZJAB3, 2005JSAB1 and 2005LNAB4) contained neither *bla*_OXA-23_ nor other known carbapenemase-encoding genes. Mutations in *oprD* have been associated with imipenem resistance in *A. baumannii*^20^. However, the *oprD* sequences of the three strains were not different from other strains such that the mechanism of the carbapenem resistance in the three strains was still unknown. The situation was also observed in str. MDRAB55 from Taiwan, with an imipenem MIC of 6 mg/L. All of the analyzed strains carried *bla*_OXA-51_, which is thought to be intrinsic to *A. baumannii* and normally does not confer carbapenem resistance^21^.

**Figure 6:**
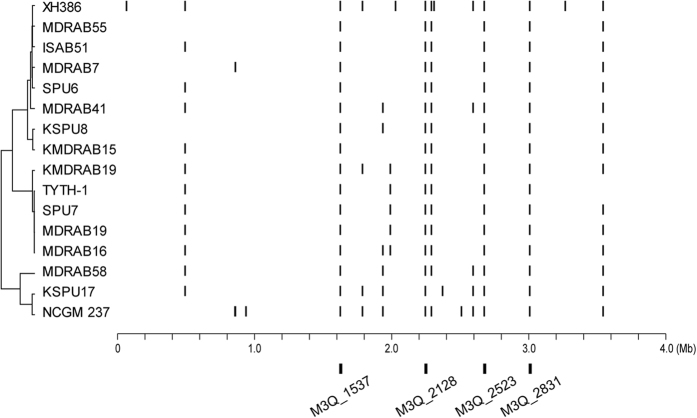
Distribution of IS*Aba1*. Bars indicate positions of IS*Aba1* insertion sites relative to the TYTH-1 chromosome. The left tree shows the phylogenetic relationship among isolates. At the bottom, the four positions in which IS*Aba1* is constantly inserted are shown.

At their upstream, *bla*_OXA-23_ is tightly linked with IS*Aba1*. Besides the function of mediating LGT, IS*Aba1* can also trigger over-expression of its downstream genes^22^. The dual role of IS*Aba1* leads us to hypothesize that this IS element prefers to insert at the upstream of those genes which would bring fitness advantage to the bacterial host. Therefore, we searched the insertion sites of IS*Aba1* for the sequenced strains. Although the pattern was similar to one another, each strain had their unique insertion sites, which were different from their close relatives (Fig. 6). This result suggested a frequent occurrence of IS insertion/deletion (indel) events after the very short divergence of these closely related strains.

Four IS*Aba1* insertion sites were commonly shared among all strains, that is, *bla*_ADC-25_ (M3Q_2831), TonB-dependent siderophore receptor (M3Q_2523), AraC-type DNA-binding domain-containing protein (M3Q_2128) and oxidoreductase (M3Q_1537). The gene *bla*_ADC-25_ encodes the AmpC cephalosporinase that can degrade a variety of cephalosporin such as ceftazidime^23^. The biological significance of the TonB-dependent siderophore receptor is also known, which is an outer membrane protein involved in iron uptake and virulence^24^,^25^. The exact roles of the latter two proteins are unclear.

### Genomic variations of AbaR-like resistance islands and class I integron

**Figure 7:**
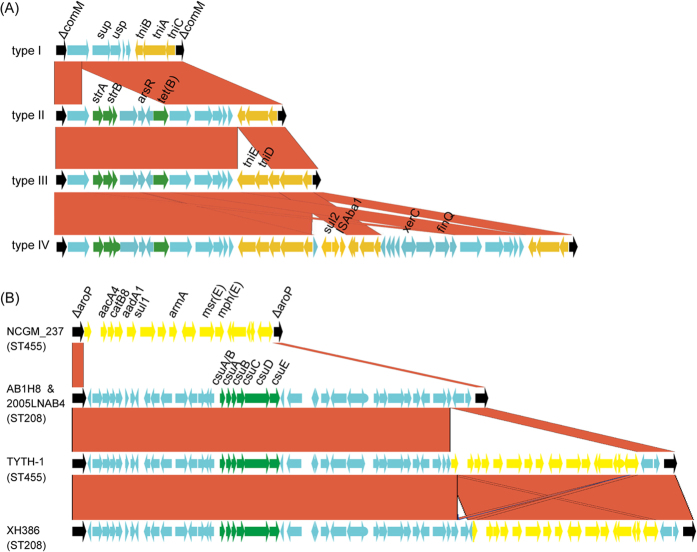
Structural variation of antimicrobial resistance elements. Panel (**A**) shows structure variation of AbaRI. Black genes represent boundary gene *comM*; green genes represent resistance genes; yellow genes represent mobile elements. Red block between different AbaRI types represent conserved genes. Panel (**B**) shows structure variation within the gene *aroP*. Black genes represent boundary gene *aroP*; yellow genes represent class I integron, green genes represent the *csu* pili operon, and red blocks between strains represent conserved genes.

AbaRI is a large collection of genes involved in antimicrobial resistance and heavy metal metabolism, which is constantly inserted at the gene *comM*^26^,^27^. Only 19 of the strains analyzed had their inserts within *comM* having been completely sequenced in this study, and four types of AbaRI were identified (Fig. 7A). The four AbaRIs shared the same backbone with several indels interspersing in it. Two indels were associated with antimicrobial resistance: one contained *sul2* and the other *strA*, *strB*, *arsR* and *tetB*. While the former indel was probably mediated by IS*Aba1*, the latter contained no adjacent IS or direct repeats so that it was likely to be mediated by recombination. The AbaRI type was inconsistent with the phylogenetic relationship. For example, within SubClade 2, str. XH386 carried type IV AbaRI, str. AB1H8 carried type II AbaRI, and str. 2011HNAB1 and 2005JSAB1 carried type III AbaRI (Figs 2 and 7A). Such inconsistency indicated frequent variation occurring at this locus, mediated either by IS insertion or by recombination.

Class I integron, which is usually inserted within the gene *aroP*, is another important vector of antimicrobial resistance genes in *A. baumannii*. In our study, the inserts within *aroP* were completely sequenced in five strains, and three contained class I integron (Fig. 7B). The three identified integrons were identical to one another, all containing multiple resistance genes, such as *aacC1*, *aadA1* and *aacA4*. A 6-kb *csuE* operon was also found within *aroP*. Although this operon is involved in pilus and biofilm formation^28^, its function seems dispensable because it is absent in seven strains examined in this study (Fig. 5). Similar to AbaRI, the structure within *aroP* exhibited great variability (Fig. 7B). For example, str. XH386 and AB1H8 differed by less than 100 SNPs in their core genomes, but the former contained class I integron while the latter did not.

## Discussion

In this study we applied WGS to investigate an outbreak of *A. baumannii* bacteremia and the phylogenetic relationship of the widely disseminated strains in East Asia. Although these strains were too close to be differentiated by MLST, a high degree of genomic plasticity was observed in their genomes. In particular, WGS separated ST455 into two independent clades, whereas strains belonging to different STs showed a closer relationship (Fig. 2). Other studies have revealed that WGS is more accurate than conventional techniques, such as MLST and pulsed field gel electrophoresis (PFGE), in discriminating among alternate transmission scenarios during outbreaks of MDR *A. baumannii*, carbapenem-resistant *Klebsiella pneumoniae* and carbapenem-resistant *Enterobacter cloacae*^5^,^29^,^30^. These lines of evidence in conjunction with our own clearly demonstrate that WGS holds a greater discriminatory power in outbreak analysis and therefore represents a promising tool for bacterial epidemiological and evolutionary studies. In addition, this study also revealed some discrepancies between the occurrence of antimicrobial determinants and resistance phenotypes. Antimicrobial susceptibility testing demonstrated the varied susceptibility profiles within ST208 strains. *In silico* profiles using WGS data against the ResFinder database could predict laboratory resistance for only a subset of antimicrobial determinants (Fig. 5). This suggests that experimental verification is still necessary, although antimicrobial resistance databases appear useful in predicting resistance to some classes of antimicrobials in *A. baumannii*.

The core-SNP tree highlights the complexity in determining the clonality and the possible transmission routes of *A. baumannii* infection because it tends to be erroneous to deduce the phylogenetic relationship simply based on the isolation site and time. On one hand, we found that certain *A. baumannii* strains, although being collected from distant areas or over a long time span, showed nearly identical SNP profile (<50 SNPs). Such a short genetic distance would give a misunderstanding that these similar isolates originated from the same outbreak, but actually no travel history could be tracked in the corresponding patients. The phenomenon of high similarity in the genomes of international isolates was reported not only in *A. baumannii* in this study but also in other pathogens such as *Staphylococcus aureus*^31^. On the other hand, most of the analyzed Taiwan strains, even those belonging to the same ST and collected from the same hospital, could hardly be classified into the same subclades. Thus the bacteremic cases in CGMH did not belong to a single-clone-caused outbreak.

In general, the shorter the genetic distance between strains, the less indel events occur^32^. Therefore the disseminated isolates were expected to possess identical gene content. However, frequent genomic variation was observed even inside the same clade of *A. baumannii*. Resistance genes are prone to be the target accumulating such variations, suggesting that the resistance elements respond actively to the selection pressure in the hospital setting. Interestingly, we found that Tn*2009* was predominant but also restricted to Mainland China. To our knowledge, the earliest report of Tn*2009* in China is the year 2011, and yet no publications have reported the occurrence of Tn*2009* in East Asian countries other than China^33^,^34^,^35^. One possible explanation for this observation is the recently described plasmid pABTJ1^36^ (pAZJ221)^34^, which appears to be the major driving force for the spread of Tn*2009* in China. The replicase gene of the plasmid cannot be classified as any of the previously defined replicons, though it shares ~67% nucleotide identity with *repAci6*^37^. Like Tn*2009*, this plasmid has not been reported in any country other than China. In this regard, we hypothesize that a genetic or ecological barrier may exist, preventing the transmission of Tn*2009* by the conjugative plasmid. However, this hypothesis needs further experimental verification.

Previously, several studies described that *A. baumannii* hospital outbreaks can be polyclonal, and a variety of recombination and LGT events occurred in *A. baumannii* strains and contributed to genetic diversity in the microorganism^5^,^38^,^39^. In this study, we found that one clade of ST455 presented a mosaic structure in genome (Fig. 3), which is, to our knowledge, the first report of chromosome-scale recombination in *A. baumannii*. Whether this event confers a fitness advantage and leads to the predominance of the recombinant clade is still unknown, but it is clear that co-circulation of different bacterial lineages provides a niche for the occurrence of such recombination.

In conclusion, our data shed important light on the mechanisms of the evolutionary process that contribute to the emergence and co-evolution of different *A. baumannii* lineages in East Asia, which are highly similar to each other but meanwhile exhibit significant genetic diversity. Through homologous recombination and lateral transfer of mobile elements, *A. baumannii* enhances its virulence and antimicrobial resistance that eventually benefit its survival in the nosocomial environment. The present study also highlights the importance of identifying and distinguishing the high-risk *A. baumannii* clones by the ultimate resolution of WGS. In the future, large-scale sampling across different areas and time scales is still necessary, with the aim to fully understand the evolutionary pattern of *A. baumannii* and to survey its rapid development of multidrug resistance.

## Methods

### Bacterial isolates and antimicrobial susceptibility testing

Clinical *A. baumannii* isolates were isolated between 2009 and 2013 from CGMHs in Taoyuan and Kaohsiung, Taiwan. The minimum inhibitory concentration (MIC) of antimicrobial agents was determined by E-test and interpreted according to the recommendations given by the Clinical and Laboratory Standards Institute (CLSI). The isolation site and year are listed in Figs 1B and 2. The map of East Asia (Fig. 1B) was generated with R version 3.1.3 (a free software environment for statistical computing and graphics, https://www.r-project.org/).

### Genome sequencing and annotation

Genomic DNA was extracted using the Qiagen DNA Purification Kit. The genomic DNA was fragmented by ultra-sonication, and the DNA fragments were subjected to the whole-genome sequencing workflow of the Illumina HiSeq 2000 system. The derived paired-end sequence reads were obtained representing over 200-fold genome coverage. Genome assembly was carried out by CLC Genomics Workbench v8.0 (http://www.clcbio.com). The draft genome was annotated by the NCBI Prokaryotic Genomes Annotation Pipeline. The assembly information of the analyzed isolates is listed in Supplementary Table 1.

### MLST and genome-based phylogeny construction

The draft genome was aligned against seven housekeeping gene sequences using BLAST and then the aligned sequences were extracted and compared to allele profiles in MLST-OD (http://pubmlst.org/) and MLST-IP (http://www.pasteur.fr/recherche/genopole/PF8/mlst/), respectively.

The software PGAP was used for identifying conserved genes among the analyzed strains^40^. The conserved genes were aligned by using Clustal W and the alignment was concatenated by using self-developed Perl scripts. The concatenated core genome was then put into MEGA 5 software for constructing the genome-based phylogenetic tree. The number of differences substitution model was adopted and the Neighbor-joining algorithm was implemented with 1,000 bootstrap replicates. The same model was adopted for calculating pairwise distance in MEGA 5.

### Identification of IS*Aba1* insertion sites

The raw Illumina reads were aligned against IS*Aba1* sequences by the NCBI Mega BLAST program. The reads with hit to IS*Aba1* were extracted and then the IS*Aba1* sequences within the reads were masked with the Cross_match program (http://www.phrap.org). The processed reads were further aligned with the genome of str. TYTH-1 (acc no. CP003856.1), and the insertion sites of IS*Aba1* were identified.

### Nucleotide sequence accession numbers

The sequences obtained in this study were submitted to NCBI GenBank database and the accession numbers of bacterial genomes sequenced and used are shown in Supplementary Figure S1.

## Additional Information

**How to cite this article**: Feng, Y. *et al.* A glimpse into evolution and dissemination of multidrug-resistant *Acinetobacter baumannii* isolates in East Asia: a comparative genomics study. *Sci. Rep.* **6**, 24342; doi: 10.1038/srep24342 (2016).

## Supplementary Material

Supplementary Table 1
